# Crevice Corrosion Mitigation in Ti-6Al-4V DMLS Alloy via Hybrid PEO/PCL/PLA Hierarchical Coatings for Biomedical Implants

**DOI:** 10.3390/jfb17100508

**Published:** 2026-10-08

**Authors:** Shaghayegh Javadi, Enrique Martinez-Campos, Raúl Arrabal, Endzhe Matykina

**Affiliations:** 1 Departamento de Ingeniería Química y de Materiales, Facultad de Ciencias Químicas, Universidad Complutense de Madrid, 28040 Madrid, Spain; rarrabal@ucm.es; 2 Instituto de Ciencia y Tecnología de Polímeros (ICTP), Consejo Superior de Investigaciones Científicas (CSIC), C/Juan de la Cierva 3, 28006 Madrid, Spain; e.martinez.campos@csic.es; 3 Unidad Asociada al ICTP, IQM (CSIC), Grupo de Síntesis Orgánica y Bioevaluación, Instituto Pluridisciplinar (UCM), Paseo de Juan XXIII, 1, 28040 Madrid, Spain

**Keywords:** Ti-6Al-4V, additive manufacturing, crevice corrosion, plasma electrolytic oxidation, biodegradable polymer, breath figures, hierarchical coating, simulated body fluid

## Abstract

Additively manufactured implants may be vulnerable to localized corrosion within narrow gaps, yet the critical crevice geometry and the effectiveness of multilayer coatings under such conditions remain insufficiently understood. This study compares the crevice-corrosion behavior of direct metal laser sintering (DMLS) and conventionally wrought mill-annealed Ti-6Al-4V alloys and evaluates a hierarchical coating comprising a plasma electrolytic oxidation (PEO) layer, a polycaprolactone (PCL) seal, and a porous polylactic acid (PLA) breath-figure topcoat. Electrochemical testing was conducted in simulated body fluid (SBF, pH 7.4, 37 °C) using a tapered crevice cell, followed by microscopic, compositional, and topographical analyses. Both uncoated alloys exhibited passive film breakdown; however, the wrought alloy subsequently repassivated, whereas the DMLS alloy underwent sustained localized attack within the critical crevice gap of ~11.4 µm. The transpassive potentials for DMLS and wrought Ti-6Al-4V with PEO/PCL/PLA coating system were 1.32 V and 1.45 V, respectively. Despite the intrinsic porosity and interfacial gaps of the coating, no crevice-type damage was detected in DMLS Ti-6Al-4V. PCL/PLA hierarchy increased the PEO pore resistance to ~3.5 kΩ cm^2^, reduced the passive current density by ~2 times, and increased the PEO barrier layer resistance to 10^7^ Ω cm^2^.

## 1. Introduction

Additive manufacturing (AM) is a breakthrough technique that uses layer-by-layer fabrication methods such as selective laser melting (SLM) or electron beam melting (EBM) to produce complex, lightweight parts directly from digital designs. This approach reduces material waste compared to traditional machining and enables the production of intricate geometries that are difficult or impossible to achieve with conventional methods. It is especially valuable in biomedical applications, which often require customized anatomical designs of prostheses. However, the unique microstructures resulting from AM processes, such as SLM and EBM, can influence the alloy’s susceptibility to localized corrosion phenomena, including crevice corrosion [1,2].

The rapid heating and cooling cycles inherent to AM processes produce a distinctive non-equilibrium microstructure in Ti-6Al-4V, typically consisting of columnar prior-β grains containing fine acicular α′ martensite in the as-built condition [3,4,5]. The formation of α′ martensite is associated with the high cooling rates during laser-based processing, which suppress the diffusional transformation of β into the equilibrium α + β microstructure commonly observed in wrought Ti-6Al-4V [3,4,5]. Consequently, the as-built AM microstructure differs substantially from the equiaxed or bimodal α + β structures commonly obtained through conventional thermomechanical processing [5,6]. Post-processing heat treatment is therefore frequently employed to modify the metastable as-built microstructure, relieve residual stresses, and promote the decomposition of α′ martensite into more stable α + β phases [4,5,6,7]. Depending on the treatment temperature and cooling rate, heat treatment can lead to different α/β morphologies, including lamellar, Widmanstätten (basketweave) or bimodal structures, thereby significantly altering the resulting material properties [3,5,6,7]. These microstructural modifications are particularly relevant to the performance of AM Ti-6Al-4V in biomedical environments, where differences in phase constitution, grain morphology, residual stress, and surface condition may affect its corrosion response.

Research indicates that the corrosion resistance of AM Ti-6Al-4V is significantly influenced by its microstructural characteristics, with the β-phase content playing a crucial role in influencing corrosion resistance [8]. Mora-Sanchez et al. have shown that AM Ti-6Al-4V exhibits porosity and the presence of non-equilibrium phases like martensitic α′, which can compromise the integrity of the passive oxide layer and promote localized corrosion in confined spaces [9,10,11]. Additionally, the anisotropic nature of AM builds, characterized by layer-by-layer deposition, can result in directional differences in corrosion behavior [12], further complicating the material’s performance in crevice-prone medical and implant applications.

Crevice corrosion arises due to limited oxygen transport and the development of ion concentration differences within the gap between two contacting surfaces or beneath deposits. As a result, the region inside the crevice becomes acidic and enriched with aggressive ions, such as Cl^−^, leading to a significantly increased corrosion rate [13]. Since the crevice is narrow and the ionic conduction path within it is restricted, the electrical resistance of the solution inside the crevice increases and the potential drop inside the crevice becomes more severe. In these conditions, the potential of the metal surface in the inner region of the crevice can become significantly lower than that of the outer free surface exposed to the environment. If this potential drop is large enough, the metal surface inside the crevice may lose its passive state, and anodic dissolution continues at a higher rate [14].

Studies have shown that the susceptibility of titanium to crevice corrosion increases with decreasing crevice size and increasing temperature. Crevices with widths below approximately 100 µm are considered critical geometries because they intensify mass-transport limitations, oxygen depletion, chloride accumulation, and local pH reduction. In addition, increasing temperature can reduce the stability of the passive film and facilitate the initiation of localized corrosion. In many reported studies, critical behavior of titanium and its alloys has been observed at temperatures above approximately 70–80 °C [15], although the exact threshold strongly depends on alloy composition, oxygen content, solution chemistry, and crevice geometry.

In biomedical applications, very tight crevices (far below 100 µm) can form between bone plates and screws, between the stem and head of an artificial hip joint, between the implant and bone, or in regions where biofilms accumulate. Further, surgery, inflammation, infection, or hematoma formation can reduce the pH of the normal physiological environment from 7.4 to about 5.5 or even lower [16,17]. Such an environment, together with the presence of chloride ions and oxygen depletion, can provide the conditions required for breakdown of the passive film on titanium causing serious damage and titanium ion release.

Surface modification strategies aim to enhance the corrosion resistance of AM Ti-6Al-4V by providing additional barriers to corrosive agents and promoting the formation of a more stable passive oxide layer. Plasma electrolytic oxidation (PEO), also called micro-arc oxidation, is a surface treatment used particularly on light metals (aluminum, magnesium, titanium) to grow thick oxide-based coatings under high voltage involving dielectric breakdown micro-discharges in an electrolyte [1,18]. These coatings on titanium are typically hard, wear resistant, with phases like anatase/rutile TiO_2_ and often with possibilities to incorporate other oxides, particles, or bioactive elements (Ca, P, Zn, Si, etc.) [1].

Importantly, PEO coatings are porous, which is beneficial for cell adhesion and offer a possibility of drug-loading. Combining a PEO coating with a polymeric top-coat gives the advantage of an extra barrier that further improves the anticorrosion performance of the system and helps to achieve a controlled drug release [19]. However, a monolithic polymer layer may lead to the loss of the developed topography. Therefore, hierarchical hybrid coatings consisting of several layers with different micro- and nanoscale features have emerged to facilitate the tissue-implant interaction [20].

Several hybrid coating strategies have been investigated to combine PEO layers with polymeric, natural, or ceramic/inorganic components, with the aim of providing complementary protective, biological, and functional properties. PEO/polymer-based systems have been developed for biomedical applications, including drug delivery, antibacterial activity, antifouling performance, and surface functionalization [21,22,23,24,25]. For example, a doxycycline-loaded oxide–polymer coating was developed on a PEO-treated Ti–Mo alloy, demonstrating the potential of combining a porous oxide layer with a polymeric phase for drug delivery and antibacterial applications [21]. Similarly, hyaluronic-acid-based antimicrobial coatings have been deposited on PEO-modified titanium surfaces to improve their antifouling and biological properties [22], while drug-loaded poly(sebacic anhydride) layers have been used to functionalize PEO-treated Ti–15Mo surfaces while retaining the functionality of the porous oxide structure [23]. Hybrid PEO/polymer systems have also been investigated on biodegradable magnesium alloys, such as PEO/PLA coatings on ZK60, where the polymer layer was used to seal the porous PEO coating and improve corrosion protection [24]. In another titanium-based application, a porous PEO layer combined with a drug-loaded polymer layer was developed on titanium bone wedges to provide antibacterial functionality and biocompatibility [25].

In addition to polymer-based systems, PEO coatings have been combined with ceramic or inorganic layers to enhance corrosion protection and biological performance. A PEO/sol–gel composite containing graphene oxide was developed on Ti-6Al-4V, where the secondary layer filled the micropores of the PEO coating and improved its corrosion and tribocorrosion resistance [26]. Similarly, a duplex PEO-based coating incorporating Zn on Ti-6Al-4V was developed to provide an osteoconductive surface and promote cell growth [27]. Hybrid ceramic/ceramic coatings have also been investigated on magnesium alloys, demonstrating the broader applicability of combining PEO with secondary protective layers such as hydroxyapatite for improving corrosion resistance in simulated physiological environments [28]. Collectively, these studies demonstrate that the incorporation of a secondary polymeric, natural, ceramic, or inorganic layer can complement the porous PEO structure and provide additional functionality beyond that achievable with a single PEO coating.

Among the different fabrication methods, the breath figure (BF) approach has been introduced as an effective way to develop hierarchical hybrid coating systems. It provides a useful route for preparing polymer layers through the self-assembly of condensed water droplets, which leads to controllable micro- and nanoscale porosity [29,30,31,32]. Previous studies have shown that the porous structure of BF coatings in hierarchical hybrid systems is useful for controlled drug release, while the porous polymer layer also provides a suitable surface for cell attachment and growth.

It is also worth noting that cell growth has been observed throughout the three-dimensional porous architecture of BF-based coatings [19,32]. While it is a highly positive phenomenon from the implant-tissue interaction point of view, it also raises concerns with respect to the possibility of crevice corrosion of AM Ti-6Al-4V alloys with multilayer hybrid coatings. In such systems, the layered structure may create interfacial gaps or crevice-like defects between the coating layers, which can provide suitable conditions for the initiation and propagation of localized corrosion. The novelty of this work is based on the fact that crevice corrosion behavior of titanium substrates protected by multilayer hybrid coatings has not yet been systematically studied. Importantly, there is still no comprehensive understanding of the critical crevice size in additively manufactured alloys in the current literature. Therefore, the present study aims to fill these research gaps by evaluating the corrosion behavior of additively manufactured Ti-6Al-4V with and without a hierarchical hybrid ceramic/polymeric coating system in a specially designed cell that features controlled crevice-forming conditions.

## 2. Materials and Methods

### 2.1. Specimen Preparation

The reference material consisted of a 0.5 mm-thick Ti-6Al-4V Grade 5 sheet, which was wrought and subjected to conventional wrought processing (hot-rolled and mill-annealed), hereafter designated as ConvTi64. Additively manufactured Ti-6Al-4V Grade 5 specimens, referred to as AMTi64, were produced using laser powder bed fusion (L-PBF) at Fundación Idonial (Gijón, Spain) with an EOS M280 system (Krailling, Germany). Comprehensive details regarding the powder feedstock and printing parameters have been documented in previous studies [1,2].

As-built AMTi64 specimens underwent stress-relief treatment at 300 °C for 2 h. Subsequently, a heat treatment was applied at 750 °C for 3.5 h to transform the corrosion-prone acicular α’ microstructure into a lamellar α + β structure and improve the overall corrosion resistance of the alloy [9,33]. After the heat treatment, specimens measuring 30 × 10 × 2 mm were sectioned along the XY plane.

All samples were ground using SiC papers up to P1200 grit to achieve a smooth and flat surface. They were then etched in a Keller-type solution composed of 95.5 mL H_2_O, 2.5 mL HNO_3_, 1.5 mL HCl, and 0.5 mL HF for 180 s. Etching the samples reveals the microstructure and grain boundaries of the alloy. This procedure removes the mechanically deformed layers resulting from polishing and exposes the true structure. Additionally, etching increases the contrast between phases, enabling more accurate microscopic observation [34].

### 2.2. Plasma Electrolytic Oxidation

The samples were connected to a copper wire to establish electrical contact during the process. The surface of the samples was painted with electrically insulating lacquer No. 45 (MacDermid Enthone, Waterbury, CT, USA), leaving exposed on each side a total working area of 4 cm^2^. The PEO electrolyte was an aqueous solution containing Ca, P, Zn, Mg, and Si as bioactive species, with the following composition (reagents supplied by Merck, Darmstadt, Germany): Ca(CH_3_COO)_2_·10H_2_O 15.4 g·L^−1^, NaH_2_PO_4_·2H_2_O 3.9 g·L^−1^, Na_2_EDTA·2H_2_O 20.5 g·L^−1^, ZnO 2 g·L^−1^, Na_2_SiO_3_·5H_2_O 3 g·L^−1^, MgSO_4_ 3 g·L^−1^, pH 5.0.

The PEO process was carried out using a 2 kW DC power supply (EAC-S2000, ET Systems Electronic, Altlussheim, Germany) that applied an AC square waveform with a 50% duty cycle. This signal consisted of a positive pulse of 490 V and a negative pulse of 30 V, with an effective voltage (V_RMS) of 347 V, operating at a frequency of 300 Hz. To prevent a sudden voltage and current surge, an initial ramp was applied for 60 s [1].

During the process, the effective current density (I_RMS) was limited to a maximum of 300 mA·cm^−2^. The total treatment duration for both alloys (ConvTi64 and AMTi64) was 60 s, including the initial current ramp, a condition referred to as flash-PEO [1].

The process was carried out in a double-walled glass vessel with a volume of 2 L, which was maintained at a constant temperature of 20 °C using a water circulation and thermostat system. A cylindrical mesh of 316L stainless steel was used as the counter electrode.

### 2.3. PCL/PLA Hierarchical Coatings

To produce the hybrid hierarchical coating, PEO-treated samples of both alloys were first treated in a polymeric sealing solution containing polycaprolactone (PCL) at a concentration of 20 g/L in chloroform. The sealing process was carried out using a dip-coater (ND-DC 11/1 150, Nadetech Innovations, Noáin, Spain) under room temperature conditions, at a sample immersion and withdrawal rate of 2 mm/min.

After drying, a BF polylactic acid (PLA) polymeric layer was applied on top of the PEO/PCL systems. The application was carried out in a Dycometal/ CCK 0/81 climatic cabinet (Barcelona, Spain) at constant relative humidity (>99% RH) by 4 cycles of dip-coating the specimens in a 20 g/L PLA solution in chloroform with a withdrawal speed of 120 mm/min.

### 2.4. Coating Characterization

The coating thickness was measured using an eddy current device (ISOSCOPE FMP10, Fischer Instruments, Barcelona, Spain) with an FTA3.3H probe, based on the average of 10 measurements.

Surface topography was examined using the InfiniteFocusSL system (Alicona GmbH, Graz, Austria). Surface data were recorded for 2D and 3D simulations, and topographical parameters such as S10z and Sa were analyzed using IF-Measure Suite v5.3 software.

The surface morphology and cross-section of the coatings were examined using a scanning electron microscope (SEM, JEOL JSM-6400, Akishima, Japan) and an energy-dispersive X-ray spectroscopy (EDS) system, Oxford Link (Abidong, UK). Pore size and population density were analyzed from plan-view SEM images (approximately 1100 µm^2^ per sample) using ImageJ v1.5.3t software.

For the analysis of the formed phases, X-ray diffraction (XRD) was performed using a Bruker D8 AD-VANCE A25 diffractometer (Billerica, MA, USA) with a Cu Kα radiation source (λ = 0.154056 nm). Scans were conducted over a 2θ range of 10° to 80° at a scan rate of 0.05° s^−1^, and the data were analyzed using X’Pert HighScore v3.0 software (Malvern PANalytical, Malvern, United Kingdom) in combination with the ICDD PDF5+ database (Newtown Square, PA, USA).

### 2.5. Corrosion Testing

**Figure 1:**
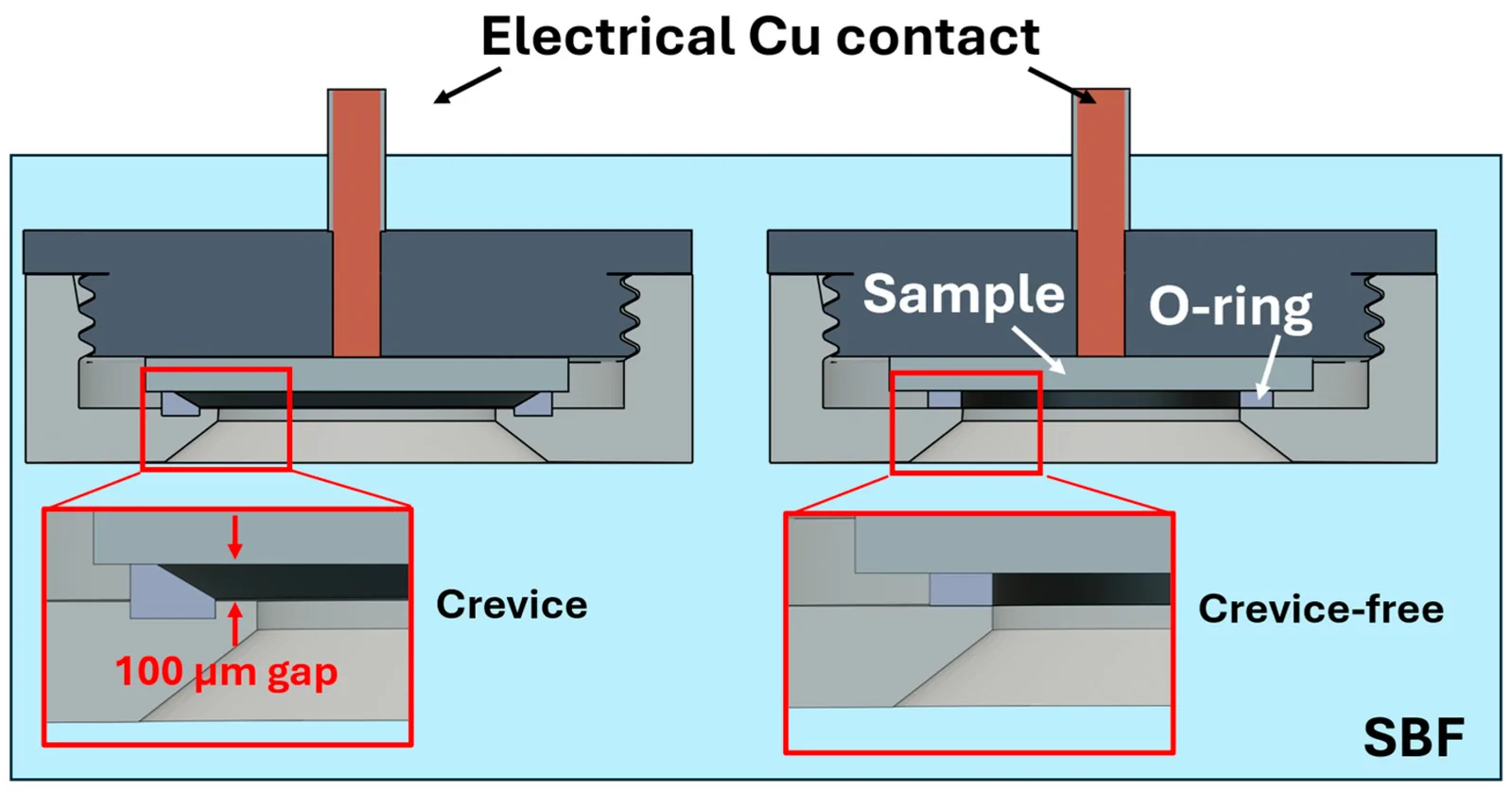
Crevice and crevice-free corrosion cartridge design.

For corrosion testing, the exposed surface area of the uncoated (control) and coated (PEO + PCL_SEAL_ + PLA_BF_) samples was limited to 0.95 cm^2^ using a specially designed crevice corrosion immersion cartridge (Figure 1). The cartridge consists of a transparent container with a built-in 100 μm-high and 3 mm-deep crevice, achieved through machining. The crevice gap was further narrowed down with the assistance of the rubber washer (O-ring) which was tapered 50% into its width. The O-ring sealed the cartridge to prevent solution leakage. The sample was positioned at the bottom of the cartridge and electrically connected via a copper contact to enable electrochemical measurements. The cell with the specimen was immersed into a double-walled electrochemical cell connected to a thermostated water bath to maintain the electrolyte temperature at 37 °C and containing 100 mL of simulated body fluid (SBF) solution. The SBF with the following composition was used as the electrolyte (reagents supplied by Merck, Darmstadt, Germany): NaCl 8.035 g·L^−1^, NaHCO_3_ 0.355 g·L^−1^, KCl 0.225 g·L^−1^, K_2_HPO_4_·3H_2_O 0.231 g·L^−1^, MgCl_2_·6H_2_O 0.311 g·L^−1^, CaCl_2_ 0.292 g·L^−1^, Na_2_SO_4_ 0.072 g·L^−1^. This formulation corresponds to the c-SBF (i.e., *c* formulation) reported by Oyane et al. [35], sans the TRIS buffer, in order to avoid the influence of TRIS on the in vitro corrosion behavior [14,36].

Potentiodynamic polarization (PDP) measurements were conducted using a Gamry Interface 1010E Potentiostat/Galvanostat/ZRA (Gamry Instruments, Warminster, PA, USA), with an Ag/AgCl reference electrode and a Pt counter electrode with an area of 1 cm^2^ after stabilizing the open circuit potential (OCP) for 1 h. Potentiodynamic polarization curves were recorded between −0.15 V and 3.0 V with respect to the OCP at a scan rate of 0.5 mV/s. In all corrosion experiments at least two samples per condition were tested, performing up to three or four repetitions, when necessary, achieving the repeatability with the relative standard deviation (RSD) of ≤8% for passive current density. Statistical analysis was done using a GraphPad *t*-test calculator (Dotmatics, Boston, MA, USA, https://www.graphpad.com/quickcalcs/ttest1/, accessed on 1 October 2026) based on an unpaired *t*-test, with a confidence interval of 95% (*p* < 0.05). Electrochemical impedance spectroscopy (EIS) was carried out applying a 10 mV sinusoidal perturbation with respect to the OCP from 10^5^ to 0.01 Hz. The spectra were fitted using ZView v3.1c software, with chi-squared values for the goodness of fit below 0.01 and parameter errors under 5–10%.

## 3. Results

### 3.1. Substrate Characterization

**Figure 2:**
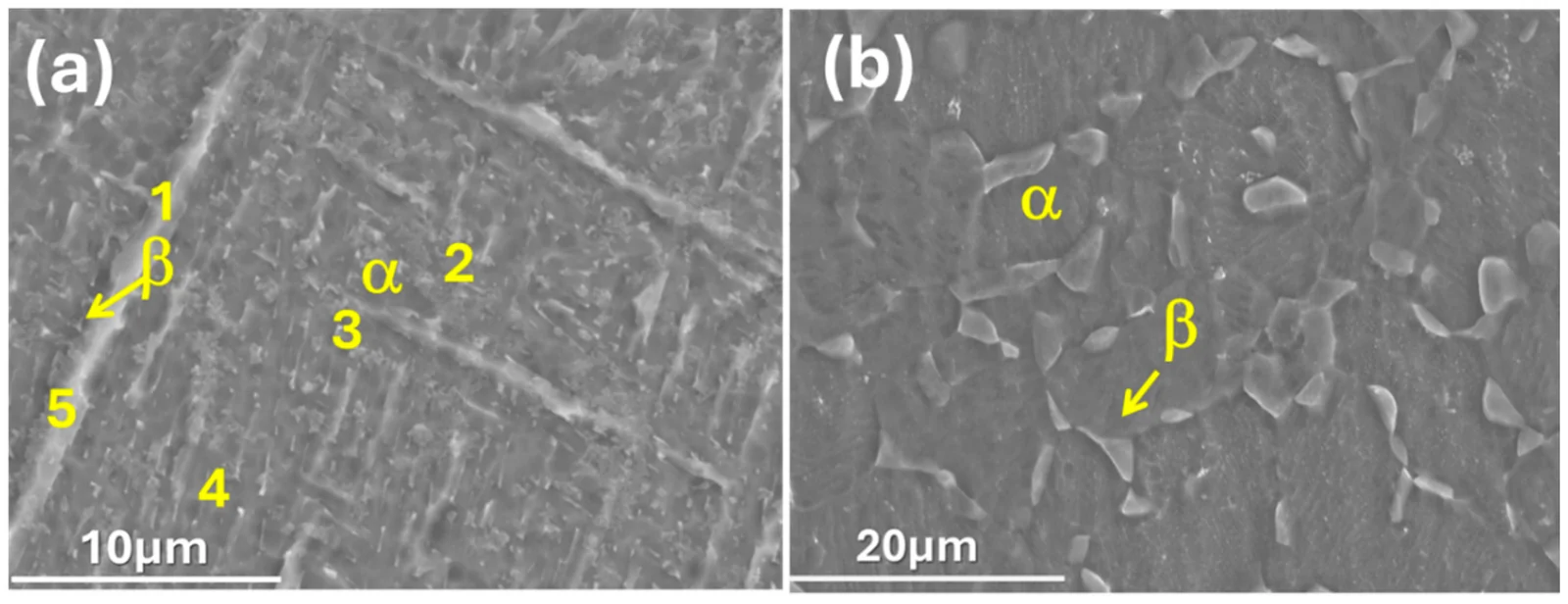
Plan view of secondary electron micrographs of etched AMTi64 (**a**) and ConvTi64 (**b**) alloys with specified locations of EDS analysis shown in Table 2.

Figure 2 presents the SEM examination of the microstructure of the ConvTi64 and AMTi64 alloys. The AMTi64 alloy exhibits a lamellar α + β morphology (Table 1) which is the result of the applied post-heat treatment. In contrast, the ConvTi64 displays coarser α grains with β phase distributed between the α grains. EDS analysis of the substrates revealed no major compositional variation between the alloys. However, traces of Si, Mg and Fe were detected in AMTi64 alloy. These minor impurities may shift the dielectric breakdown threshold and alter the initial PEO discharges [2,12,37].

### 3.2. PEO Coatings

The electrical response of PEO process on ConvTi64 and AMTi64 alloys was comparatively discussed in our previous work [2], which showed that minor differences in the oxidation behaviour exist only in the pre-breakdown region, i.e., in the first 30 s of the treatment.

**Figure 3:**
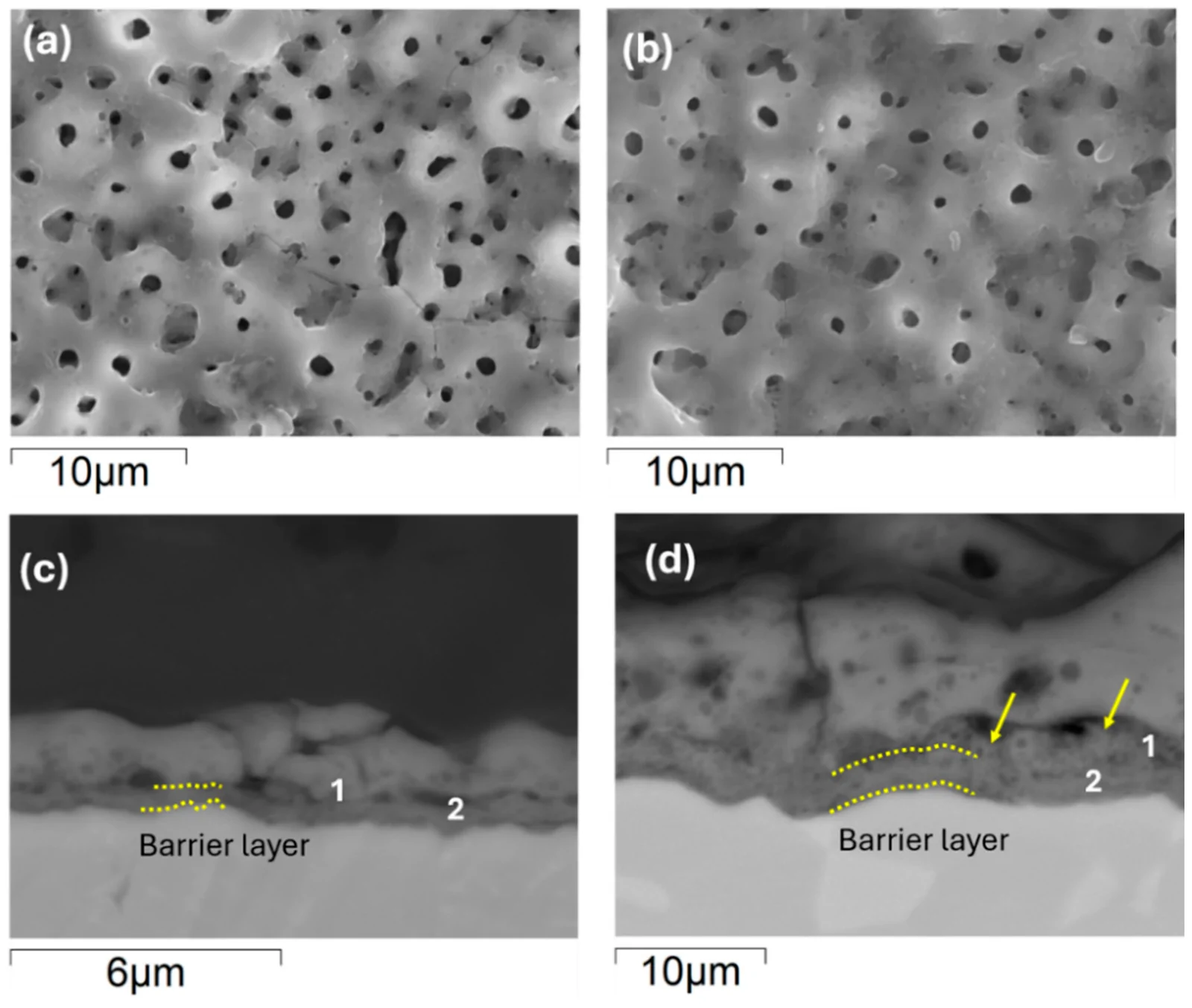
Plan view (**a**,**b**) and cross-sectional (**c**,**d**) coating microstructure of (**a**,**c**) AMTi64 and (**b**,**d**) ConvTi64 alloys after 60 s of PEO treatment. Numbers correspond to EDS analysis in Table 3. Arrows point at local thickening of the barrier layer.

The SEM image analysis (Figure 3a,b, Table 2) showed that the coating formed on the AMTi64 substrate after 60 s of PEO treatment possessed a higher pore population density but smaller average pore size and lower overall porosity (AMTi64-60s: density ≈ 1.32 × 10^6^ mm^−2^, size ≈ 0.8 µm, porosity ≈ 9.7%), whereas the coating on ConvTi64 exhibited larger pores and higher porosity (ConvTi64-60s: density ≈ 0.90 × 10^6^ mm^−2^, size ≈ 1.1 µm, porosity ≈ 13.1%). This trend is consistent with earlier onset of sparking and, possibly, higher local temperatures on the conventional substrate, which promote the formation of coarser pores, while the AMTi64 substrate, with more uniformly distributed sparks, yields a finer structure [2]. The coating porosity is crucial for the subsequent PCL sealing process and for controlling penetration behavior in the following hybrid coating stages [19].

The EDS analysis of both coatings (Table 3) confirmed the effective incorporation of electrolyte-derived species (Ca, P, Si, Mg, Zn, and S). The ConvTi64-60s sample showed a slightly higher enrichment in Ca and P, which may reflect a longer plasma exposure due to earlier spark initiation. Cross-sectional EDS profiles also revealed a compositional gradient typical for PEO coatings: the concentration of electrolyte-derived elements increased toward the outer surface, while Ti and O dominated near the inner layers. The phase composition of the coatings, reported elsewhere [2], disclosed no Ca- and P-based compounds, both coatings containing predominantly anatase, amorphous material and small amount of rutile.

The cross-sectional SEM images in Figure 3c,d further clarify the internal structure of the PEO layers. In both substrates, a thin and relatively compact barrier layer is visible directly above the Ti-6Al-4V substrate (indicated by the yellow dashed line). This inner layer is continuous and contains nanometric-size pores and voids compared with the outer region, which is consistent with other reports on the TiO_2_ barrier layer for PEO coatings [38]. On the AMTi64 substrate (Figure 3c), the barrier layer appears comparatively uniform in thickness, supporting the view that the discharge activity is more evenly distributed.

In contrast, the conventional substrate (Figure 3d) exhibits local thickening of the barrier layer (yellow arrows) which appears to expand into the so-called pore band. These features are characteristic of internal discharges occurring within cavity regions and suggest that discharge channels can locally erode and re-solidify the material down at the vicinity of the barrier layer [39,40]. Such internal discharges may explain the lower surface pore population density and larger pore size observed in ConvTi64-60s plan-view images.

### 3.3. Hierarchical PCL/PLA Coatings

**Figure 4:**
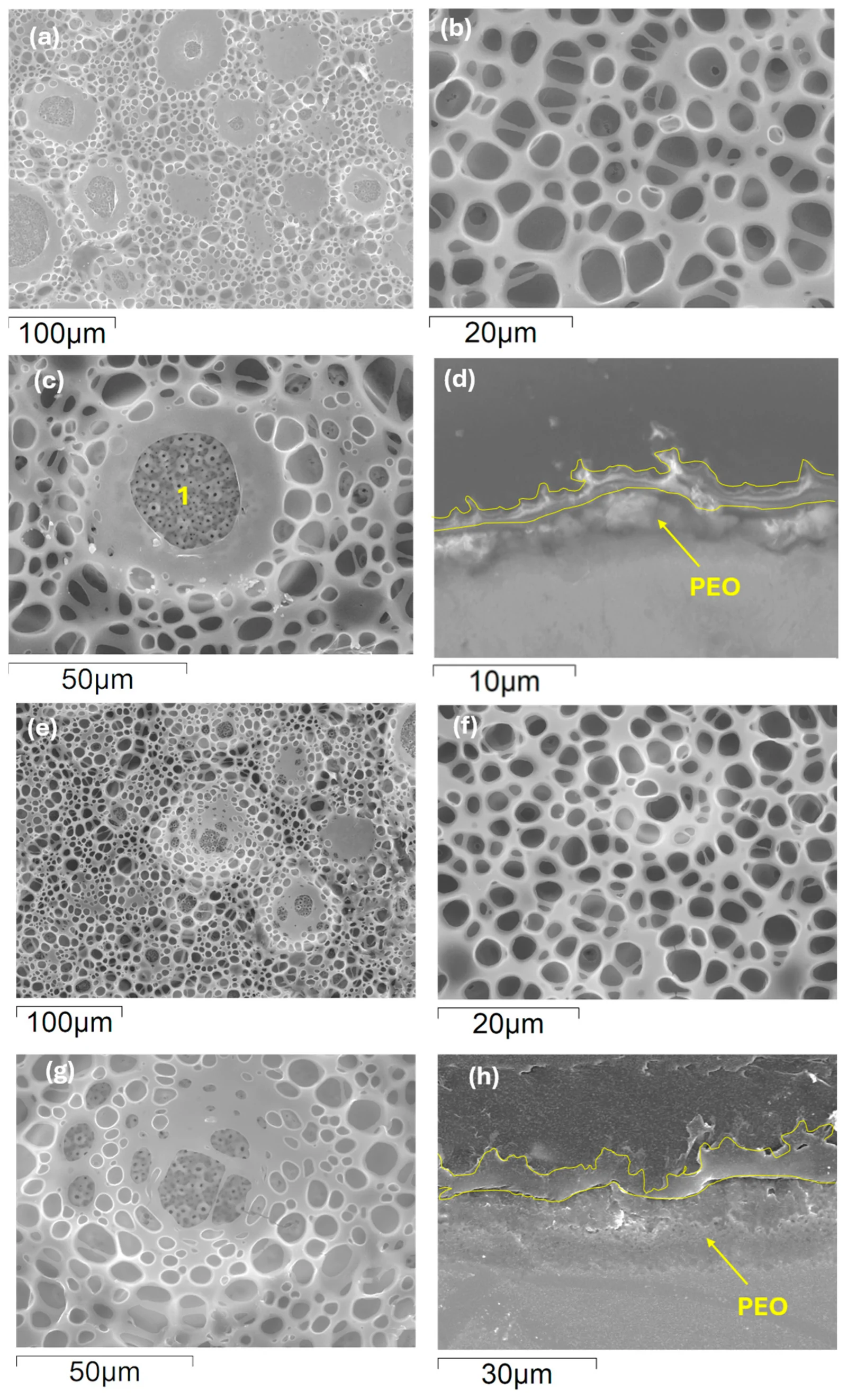
Plan view (**a**–**c**,**e**–**g**) and cross-sectional (**d**,**h**) secondary electron micrographs of PEO + PCL_SEAL_ + PLA_BF_ coating on ConvTi64 (**a**–**d**) and AMTi64 (**e**–**h**) Ti-6Al-4V alloys. Number 1 correspond EDS analysis in Table 4. The boundaries between the PEO and the PCL_SEAL_ + PLA_BF_ layers and the coating/embedding resin interfaces are indicated with yellow lines (**d**,**h**).

Figure 4 shows the microstructural characteristics of the coating system applied on the AMTi64 and ConvTi64 alloys. In both cases, a PEO oxide coating was first sealed by a thin PCL layer, upon which a porous PLA layer was subsequently formed via the BF method.

The surface morphology of the coating was generally similar in both alloys, exhibiting a characteristic BF pattern (Figure 4a,c). In both cases, the polymer surface contains quasi-spherical and partially interconnected pores with a relatively ordered distribution, which are formed through water droplet condensation on the polymer film during solvent evaporation, followed by droplet removal after drying [19,41]. In addition to the fine BF pores, with diameters ranging from approximately 1 to 6 µm on the ConvTi64 substrate and from 1 to 5 µm on the AMTi64 substrate (Figure 4b,f), occasional larger pool-like openings were also observed. The diameters of these larger openings ranged approximately from 20 to 35 µm on the ConvTi64 substrate and from 12 to 25 µm on the AMTi64 substrate (Figure 4c,g). EDS analysis (Table 4) indicated that a thin PCL layer was present inside the large opening depicted in Figure 4c, as evidenced by its elevated carbon content compared with the typical value of <20 at.% measured on stand-alone PEO specimens carbon-coated for SEM examination. Overall, despite the tendency toward somewhat larger pores on the ConvTi64 substrate, the BF layers formed on both substrates exhibited broadly comparable pore-size ranges and the same overall hierarchical porous morphology, allowing a comparative assessment of their protective behavior.

The cross-sectional images (Figure 4d,h) confirm the multilayered structure of the hybrid coating, disclosing the PEO, PCL, and PLA layers. In both alloys, the thickness of the hierarchical polymeric part (sealing layer plus BF layer) varied within 5–15 μm, depending on the local height of the pore walls.

### 3.4. Corrosion Tests

**Figure 5:**
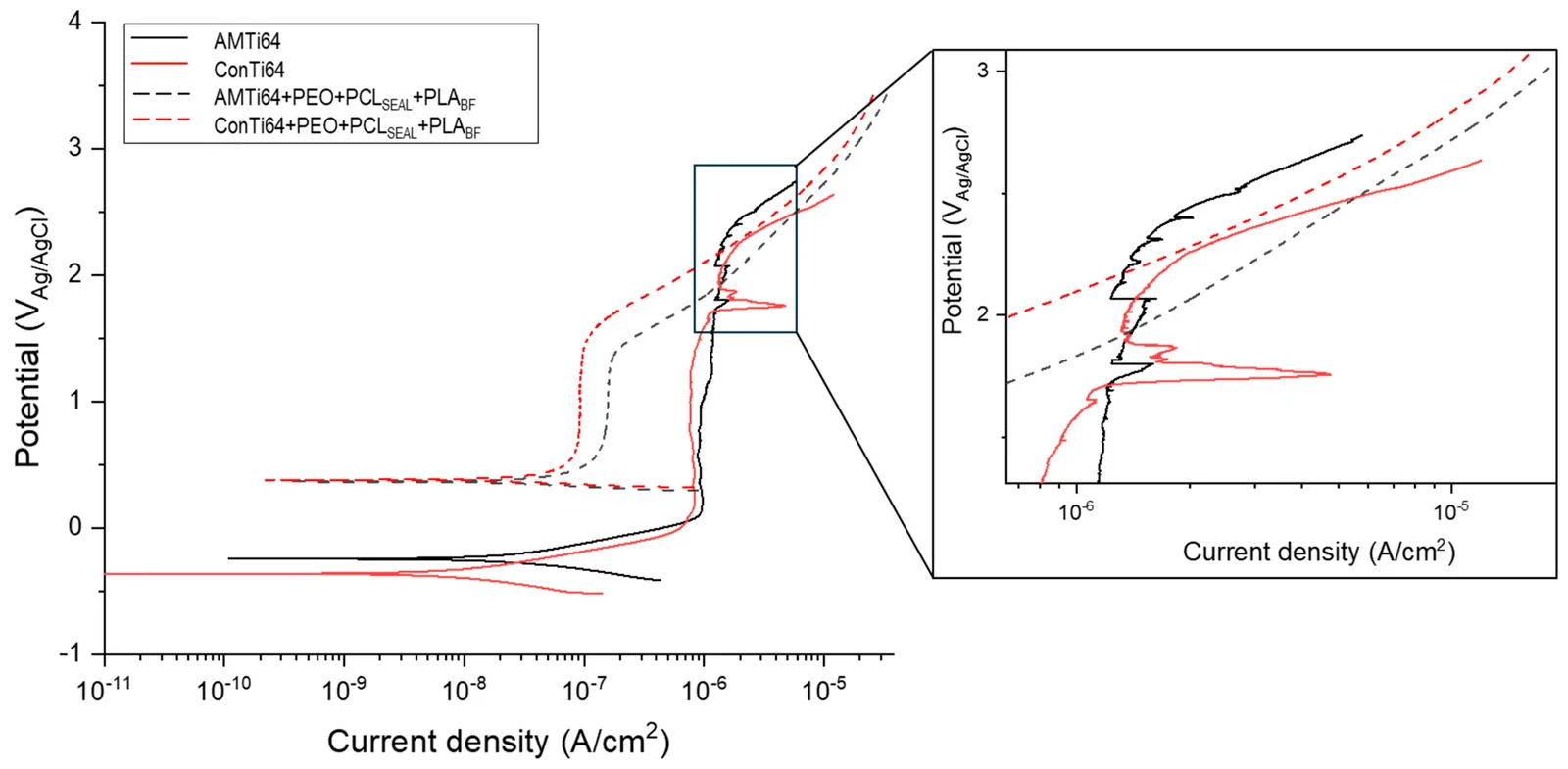
Potentiodynamic polarization curves of ConvTi64 and AMTi64 alloys without and with PEO + PCL_SEAL_ + PLA_BF_ coating obtained in a crevice cell.

For the bare substrates, the potentiodynamic curves (Figure 5) diverge in terms of corrosion potential, E_corr_, and passive current density, i_pass_ (Table 5). The corrosion current density is not of interest in this case, since it represents titanium oxidized in the entire exposed sample area and not the metal loss in the confined area of the crevice. The i_pass_ in AMTi64 is higher to begin with and exhibits a step-rise at ~1.0 V, reaching 1.2 μA/cm^2^. In both alloys, the breach of passivity and localized corrosion attack commence at E_crev_ = 1.70 V (Figure 5, inset), manifested by current rise. In ConvTi64 alloy the current peaks at 4.75 μA/cm^2^ and drops, which is consistent with repassivation. Second steady current rise from ~2.2 V is attributed to transpassive behavior (meaning that the oxide films exhibit some electrical conductivity [42,43]. Conversely, in AMTi64 alloy, several fluctuating current surges are observed (Figure 5, inset), so that even the transpassive region of the polarization curve appears serrated. This behavior suggests incomplete repassivation and a higher tendency for sustained localized attack under crevice conditions.

In alloys with full hierarchical hybrid coating system, the corrosion potentials are ~600 mV and ~740 mV more noble and passive current densities are stable and nearly one order of magnitude lower than in respective bare substrates (Figure 5 and Table 5). The transpassive region for coated samples is smooth, commencing at 1.32 V and 1.45 V for AMTi64 and ConvTi64 alloys respectively.

This difference indicates that the onset of transpassive behavior occurred at a lower anodic potential for the AM condition. Nevertheless, both coated alloys exhibited stable passive behavior over a broad potential range, with substantially lower i_pass_ values compared with their respective uncoated substrates (Table 5). The ~0.13 V difference in transpassive potential may reflect the influence of the underlying substrate microstructure. In particular, high density of α/β grain boundaries in AMti64 would result in a greater electrochemical activity of the alloy and earlier transition to transpassivity.

**Figure 6:**
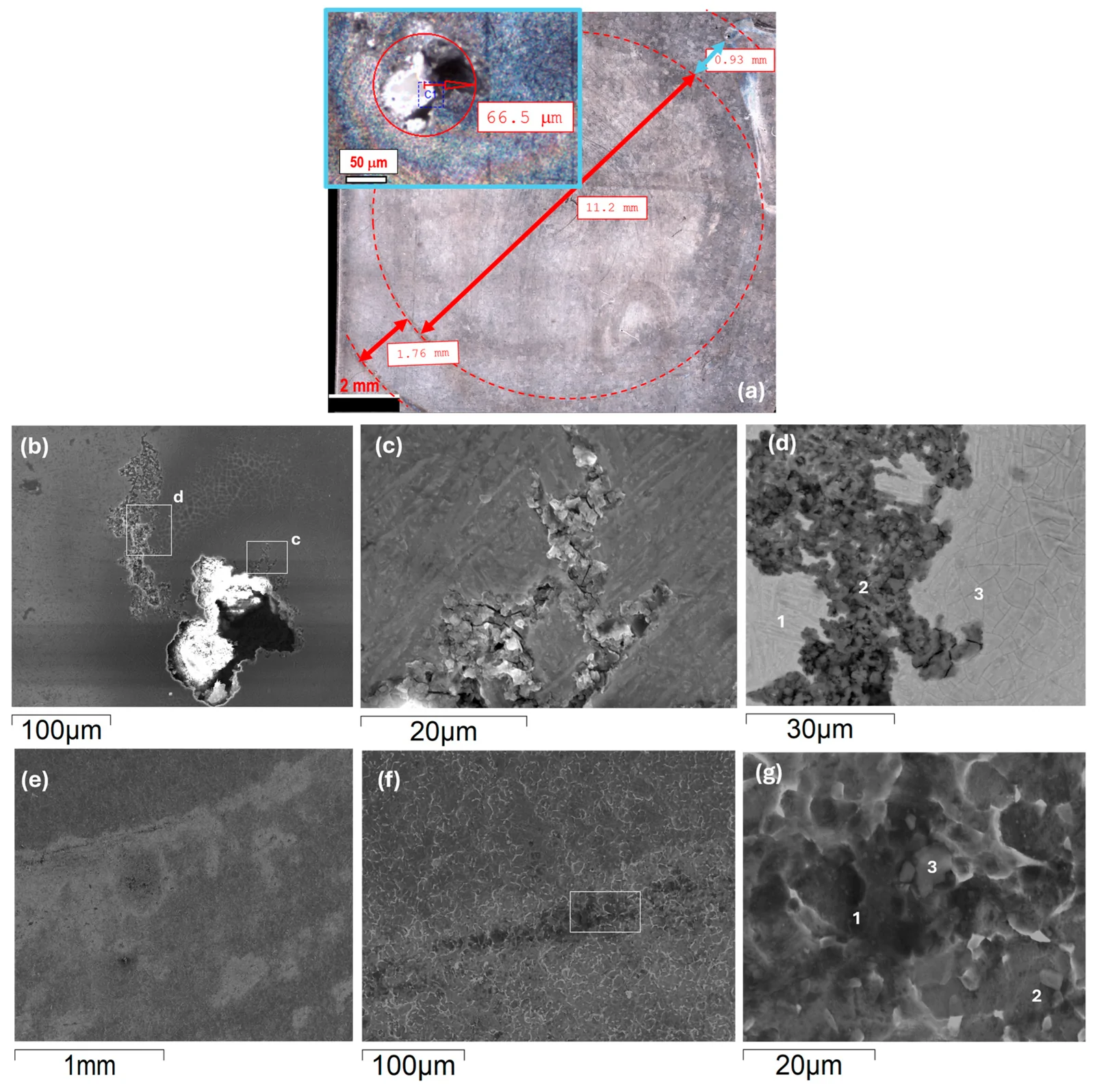
Optical (**a**) and SEM (**b**–**g**) micrographs of crevice corrosion morphologies in AMTi64 (**a**–**d**) and ConvTi64 (**e**–**g**) following potentiodynamic polarization in a crevice cell. Numbers correspond to EDS analysis in Table 6. The dashed circle in (**a**) indicates the exposed area delimited by the O-ring with an effective radius of ~5.6 mm and a width of ~1.76 mm. The inset and blue arrow in (**a**) depict the size and location of the pit with respect to the exposed area. Locations of (**c**,**d**) are marked in (**b**) with rectangles; (**g**) is magnified view of the area marked in (**f**). The (**b**,**c**,**e**–**g**) are secondary electron images and (**d**) is backscattered electron image.

Figure 6 and Table 6 present post-corrosion optical and scanning electron microscope examination results of the uncoated samples tested in the crevice cell. The AMTi64 alloy developed at least one well-defined and stable pit inside the crevice region. Large, localized damage (visible even with the naked eye) was observed under the O-ring, at 0.93 mm distance from its inner diameter contour (Figure 6a).

Upon closer inspection (Figure 6b–d), the damage consisted of a >100 μm-wide pit and more shallow, branched damage area (Figure 6c,d) composed of a porous agglomeration of corrosion products. The adjacent matrix exhibits a cracked surface film (Figure 6d), point 3). Points 1 and 3 are located on the apparently less damaged matrix, whereas point 2 lies inside the dark corrosion product region. According to the EDS results (Table 6), points 2 and 3 are enriched in oxygen (>61 at.%) and contain detectable amounts of species originating from the electrolyte (Ca, P, Cl), in comparison with point 1, which appears to be covered by an intact passive film. This combination of a porous morphology and oxygen-rich, electrolyte-containing surface chemistry is consistent with a pit cavity filled with corrosion products, surrounded by a less severely attacked passive surface [44,45].

**Figure 7:**
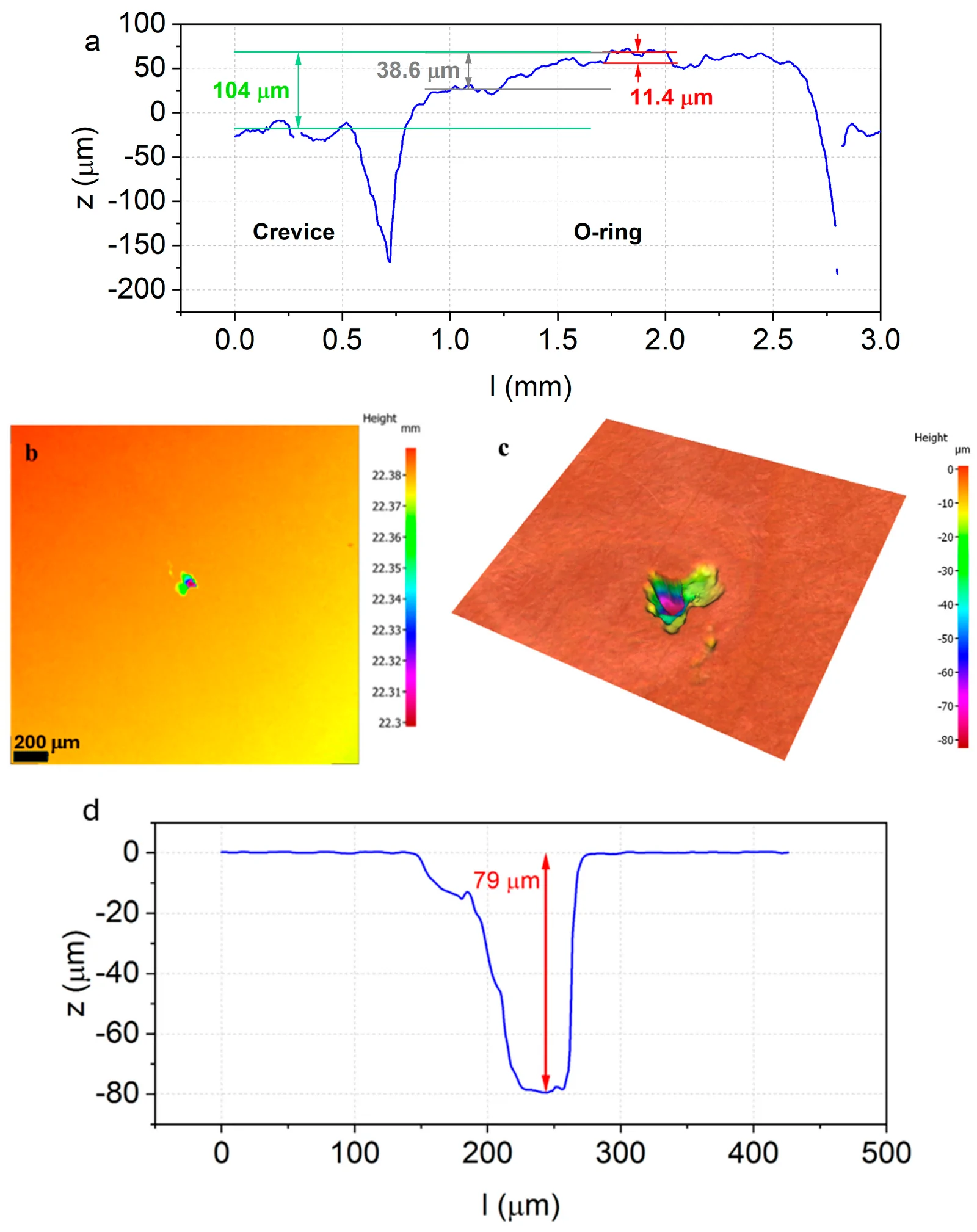
The profile of the cell crevice next to the O-ring (**a**); 2D (**b**) and 3D (**c**) topographical maps and the line profile (**d**) of localized corrosion damage in bare AMTi64 substrate.

The location of this macroscopic pit-like feature under the O-ring surface was further examined by optical profilometry in order to determine the critical crevice size. Figure 7a shows the line profile of the crevice former across the cell and O-ring surfaces. The variation in the crevice width is consistent with the experimental setup, in which the O-ring washer thickness was reduced and tapered. As a result, the local crevice confinement varied along the scanned line, achieving approximately 11.4 µm value at the deepest end of the crevice. The profile reveals a broad depressed region on the left, corresponding to the constant width of the machined crevice (3 mm-deep according to the cell design), and a smaller, tapered depression on the right associated with the O-ring contact. The largest local height difference between the specimen surface and the machined crevice roof is ~104 µm, while a smaller step of ~38.6 µm marks the beginning of the tapered section within the O-ring. The narrowest gap between the specimen surface and the O-ring is ~11.4 µm and is ~0.7 mm away from the edge of the O-ring. Because the spacer thickness was not uniform, the measured values should be interpreted as local geometrical features rather than as a single well-defined nominal crevice gap for the whole assembly.

Nevertheless, the comparison of the pit location of 0.93 mm inside the O-ring (Figure 6a) and the length of the tapered crevice profile (Figure 7a) suggests that pitting initiated at the deepest and narrowest local confinement, i.e., critical crevice size smaller than 38 µm controls the corrosion attack in AMTi64 alloy.

Figure 7b presents the plan-view height map of the pitted area (as per location in Figure 6b). Aside from the fine roughness characteristic of the additively manufactured surface, the scanned region is topographically fairly uniform, except for one clearly defined depressed feature near the center. This suggests that metal loss within the crevice region was not spatially uniform, but instead concentrated at a specific local site.

Figure 7c shows a 3D reconstruction of this feature at higher magnification. The cavity is marked by a steep depression and an uneven surrounding rim, clearly separating it from the background surface texture. The depth scale indicates that the cavity is significantly deeper than the ordinary AMTi64 surface roughness, supporting its identification as a corrosion pit. The pit depth, measured from the adjacent apparently unaffected surface to the pit bottom, is approximately 80 µm (Figure 7d). This value confirms that the observed feature is a substantial localized cavity rather than a minor surface irregularity. In combination with the global profile, the pit appears to lie in the region of strongest local confinement within the crevice-affected zone.

For the conventional alloy, much less severe localized corrosion damage was detected (Figure 6e–g). The larger-scale view in Figure 6e corresponds to the surface within the crevice footprint. It mainly shows a change in contrast but no clear macroscopic pit is present. At higher magnification, the surface consists of relatively equiaxed features revealed by shallow etching (Figure 6e,f). The corrosion morphology is uniform and without any sharply defined cavity compared to that observed in the AMTi64 alloy. EDS analyses indicate an increase in oxygen and the presence of electrolyte-derived species at points 1–3 (Figure 6g) compared with the as-polished alloy composition at point 2, confirming that these regions have experienced localized dissolution and subsequent repassivation of the oxide film. The combination of (i) only modest topographical relief at the millimetre scale and (ii) oxygen-rich but comparatively thin corrosion products at points 1–3 suggests that, in the conventional alloy, crevice corrosion progressed only through early stages of pit initiation, followed by effective re-passivation, rather than developing into a stable, propagating pit.

Taken together, these observations indicate that under identical crevice cell conditions, the AMTi64 alloy exhibits a fully developed pit in regions narrower than 38 μm, while the conventional Ti-6Al-4V alloy is capable of repassivation. These data are consistent with the corrosion test results and PDP curves (Figure 5).

Given the fact that PEO + PCL_SEALl_ + PLA_BF_ coating system presents intrinsic nano-crevices between the coating layers by design (Figure 4), it is essential to determine whether and how these might compromise the crevice corrosion resistance of the coated AMTi64 alloy. For that purpose, the potentiodynamic polarization experiments were carried out in a crevice-free cell in SBF at 37 °C and coating morphology and composition were examined post-corrosion.

**Figure 8:**
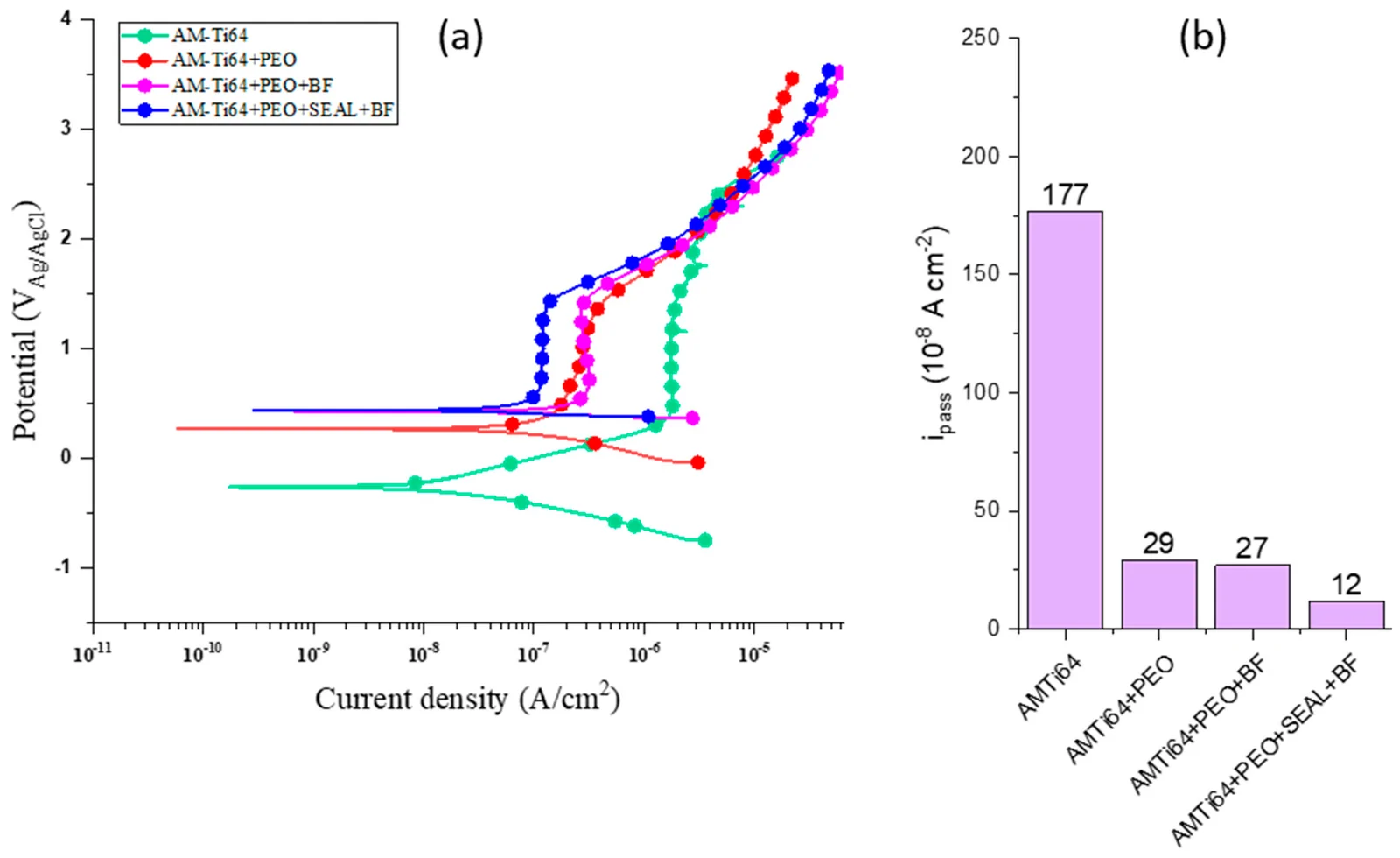
Potentiodynamic polarization curves (**a**) obtained in a crevice-free cell for AMTi64 alloy with successive layers of the PEO + PCL_SEAL_ + PLA_BF_ coating system in SBF at 37 °C and respective passive current densities (**b**).

PDP curves were obtained on the AMTi64 alloy (Figure 8a) to which ceramic and polymeric layers of the hierarchical hybrid system were applied, in order to demonstrate the contribution of each llevel of the multilayer architecture to the surface passivity (Figure 8b). The results show that ceramic PEO layer provides the primary barrier, reducing the i_pass_ by ~6 times. The open porosity of the outer PLA breath figure layer has almost no effect on the surface passivity, if applied directly onto the PEO layer. The intermediate PCL sealing layer, on the other hand, effectively blocks the pores of the ceramic coating and interfacial transport leading to a statistically significant (*p* = 0.0403) 2.25 times reduction from 0.27 μA/cm^2^ for the PEO + PLA_BF_ system to 0.12 μA/cm^2^ after application of the PCL sealing layer. PEO/polymer coating systems on Ti alloys produced by dip-or spin-coating have been reported to significantly reduce i_pass_ and broaden the passive window in chloride-containing electrolytes, in agreement with the present observations [46].

**Figure 9:**
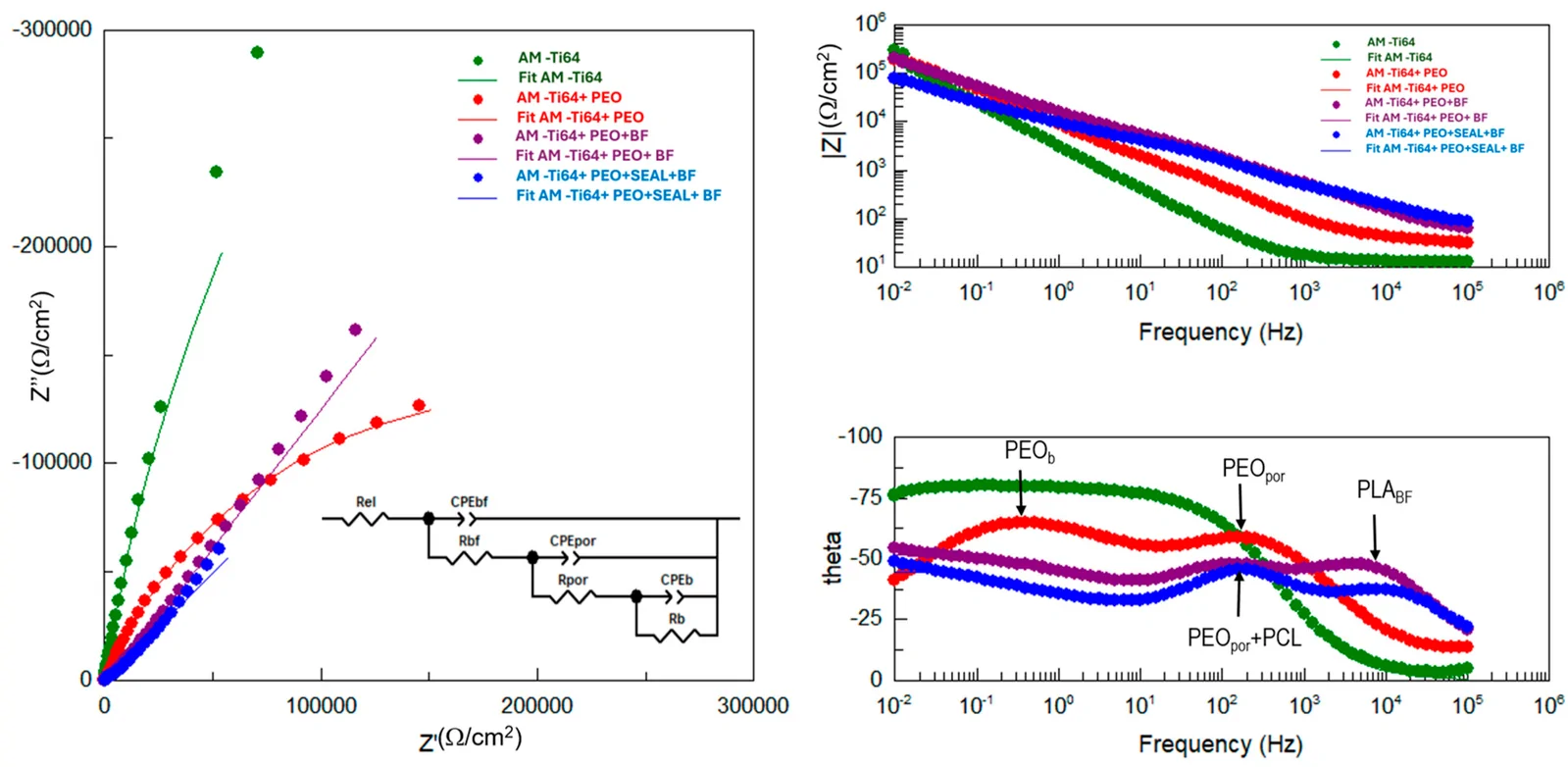
Nyquist (**left**) and Bode (**right**) plots of EIS spectra of AMTi64 alloy with successive layers of PEO + PCL_SEAL_ + PLA_BF_ coating system in SBF at 37 °C. Inset: the equivalent electrical circuit used for PEO + PLA_BF_ and PEO + PCL_SEAL_ + PLA_BF_ systems.

The EIS responses of the AMTi64 with successive elements of the full coating system obtained after 1 h of immersion in SBF at 37 °C are presented in Figure 9. The electrochemical equivalent circuits (EEC) and fitted parameters for the substrate and the PEO layer are included in Supplementary Tables S1 and S2, with the rationale for the EEC parameters previously provided in [2]. The EEC for the PEO successively coated with PCL_SEAL_ and PLA_BF_ layers includes three times constants (Figure 9, inset, and Table 7) that represent the responses of the PLA_BF_ layer (at ~10^4^ Hz), the polymer-filled PEO coating pores (at ~10^2^ Hz) and the barrier layer of the PEO coating (at the lowest frequencies). The outermost PLA breath-figure layer provides a resistance R_bf_ of 0.75–1.12 kΩ cm^2^. When the PEO pores are filled with the electrolyte, which is the case for a stand-alone ceramic coating, their resistance is typically low (25 Ω cm^2^, Supplementary Table S2), but in a hybrid system the polymer-filled pores exhibit the resistance R_por_ of 3.5–5 kΩ cm^2^ (Table 7). Interestingly, the PCL-filled pores (the case for PEO + PCL_SEAL_ + PLA_BF_ system) show lower resistance than the PLA-filled ones (the case of PEO + PLA_BF_ system). This may be related to the intrinsic differences in the dielectric properties of these two polymers, which requires a further study. Remarkably, in both hybrid coating systems the response of the PEO barrier layer is shifted to much lower frequencies (phase angle Bode plot, Figure 9) and its resistance R_b_ becomes exceptionally high (10^7^ Ω cm^2^).

**Figure 10:**
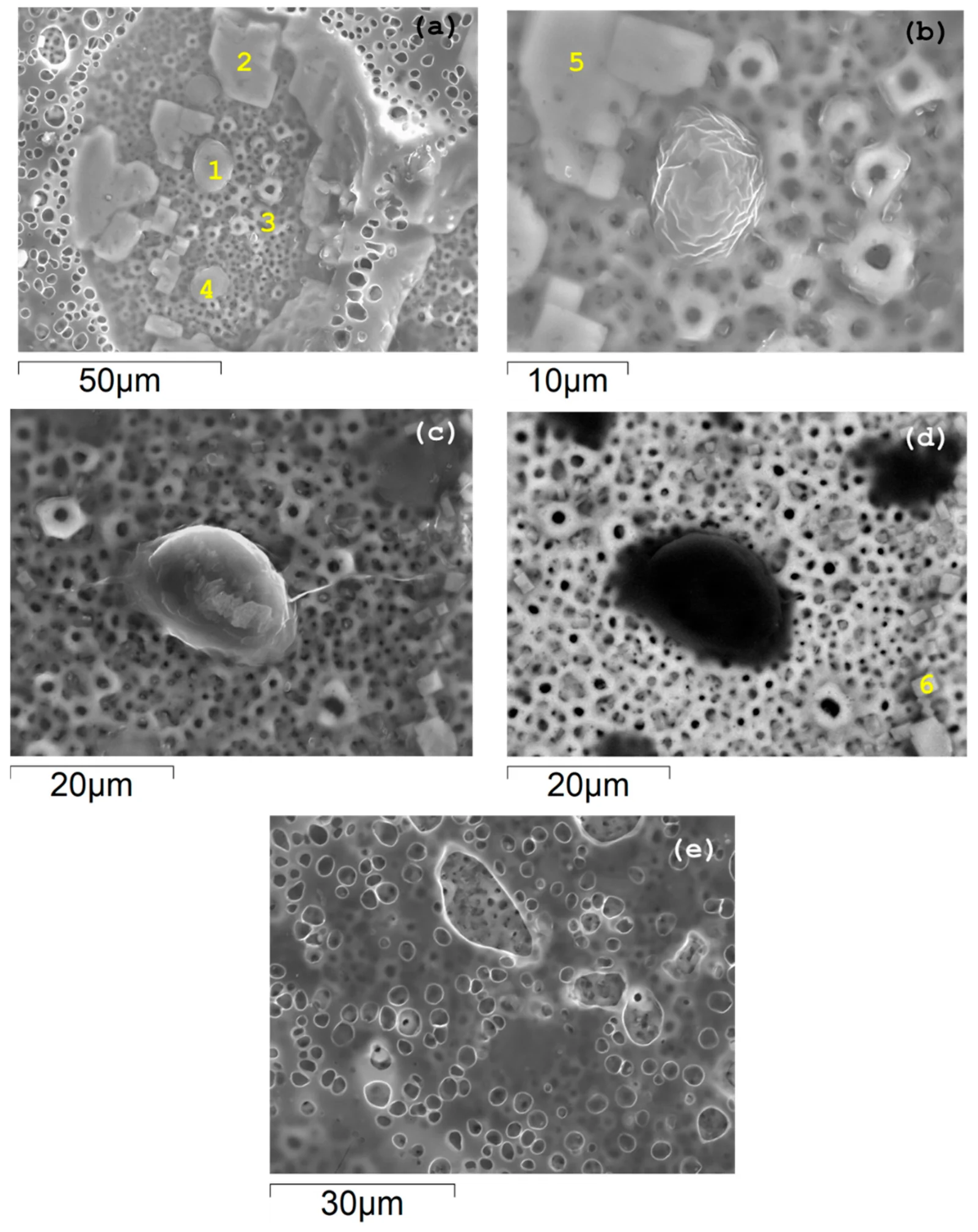
Secondary (**a**–**c**,**e**) and backscattered (**d**) electron micrographs of PEO + PCL_seal_ +PLA_BF_ coated AMTi64 alloy after potentiodynamic polarization test cell. Numbers correspond to EDS analysis shown in Table 8. (**b**) is magnification of (**a**), image taken after EDS analysis.

SEM examinations of the tested area disclosed no evidence of crevice-type attack. However, high magnification images evidenced the deposits rich in the elements of the corrosive medium formed on the surface of PEO layer under the PCL sealing (Figure 10, Table 8). The deposits, mainly consisting of polygonal NaCl crystals (locations 2, 5, 6) were found inside the large pool-like pores (Figure 10a–d), but not inside the small pores (Figure 10e), which is indicative of the presence of nano-crevices between the PLA_BF_ and PCL_SEAL_ layers in the former areas, as well as the presence of defects within the PCL_SEAL_ layer itself.

Additionally, the images reveal elliptical shapes (Figure 10b–d) inside the pool-like pores. These shapes appear to be PCL bubbles, as they are easily damaged by the electron beam at high magnification (note the wrinkled bubble in Figure 10b taken after performing the EDS analysis of the area). Polygonal Na- and Cl-rich precipitates can be seen inside and outside the bubbles (Figure 10c,d, Table 8). The bubbles could be attributed to stretching of PCL under pressure of accumulated oxygen, considering that the sample was polarized up to 3.5 V, where O_2_ evolution occurs. This is supported by high oxygen content (<80 at.%) in the corresponding locations 1 and 4 (Figure 10a, Table 8). The preferential location of the bubbles in the PCL-covered regions is consistent with the substantially lower stiffness and yield stress of PCL compared with PLA; depending on polymer grade and processing conditions, the Young’s modulus and yield stress of PCL have been reported to be approximately 5–10 and 2–3 times lower, respectively, than those of PLA [47,48]. The formation of such PCL bubbles may locally disrupt the continuity of the sealing layer and therefore affect the mechanical integrity of the coating, particularly under prolonged exposure to conditions promoting anodic polarization and gas accumulation. The lower stiffness and yield stress of PCL relative to PLA may make the PCL layer more susceptible to local deformation under accumulated gas pressure, potentially creating localized defects or pathways for electrolyte penetration. However, the absence of detectable crevice-type corrosion beneath these regions indicates that these local coating alterations did not compromise the overall protective function of the hierarchical system under the present test conditions. Previously, Nicolao-Gómez et al. have demonstrated swelling and even rupture of PCL layers during corrosion of coated Mg alloys where hydrogen gas is produced in abundance [19].

## 4. Discussion

The main purpose of the present work was to understand the crevice dimensions under which localized corrosion can be initiated and stabilized in additively manufactured Ti-6Al-4V with and without hierarchical hybrid coating system, intended for biomedical use. Although titanium is generally considered highly corrosion resistant due to its passive oxide film, localized degradation may still occur under confined geometries where mass transport is restricted and local chemistry deviates substantially from that of the bulk solution, which is especially relevant to bone-implant coupling environment [13,49]. For instance, de Damborenea et al. [50] reported localized crevice corrosion on AMTi64 associated with manufacturing-induced defects and surface imperfections that could act as crevice nucleation sites; however, the geometrical dimensions of the resulting localized corrosion features were not quantitatively reported. Mora-Sánchez et al. [1,51] focused on the occurrence, electrochemical response, and possible origins of localized corrosion, without quantitatively characterizing the crevice size.

A key outcome of this study is that crevice corrosion was observed on the uncoated substrates, both ConvTi64 and AMTi64 in SBF with a pH of 7.4 at 37 °C. This is an important result, because it shows that, for the present Ti-6Al-4V system, highly aggressive bulk conditions such as elevated temperature or initially acidic pH are not mandatory prerequisites for crevice corrosion to occur. In many previous studies, localized corrosion of titanium alloys has been discussed under more severe conditions, including elevated temperatures (e.g., 70 °C) and acidic chloride-containing environments [52,53,54,55].

The present results demonstrate that once a sufficiently tight crevice is established, the confined region itself can evolve into an aggressive micro-environment even when the external solution remains relatively mild. In the present work, the crevice profile measured after the electrochemical test revealed a gap with a dimension of approximately 11.4 µm (≈12 µm, Figure 6a and Figure 7a), indicating that geometric confinement plays an important role in destabilizing the passive state. Importantly, comparison of Figure 7a with Figure 6a,b shows that the nominal crevice gap imposed by the cell (100 µm) does not present a risk and the localized corrosion develops precisely at the site of the smallest crevice gap. This observation provides strong experimental evidence that the narrowest part of the crevice represents the critical location for corrosion initiation in the biological environment.

Although the bulk SBF solution is near-neutral, restricted oxygen renewal and mass transport within the narrow crevice establish a differential-aeration cell. The passive-state anodic process on titanium, typically represented in a simplified form as

Ti → Ti^4+^ + 4e^−^ (1)

in reality involves water and produces protons [56]:

Ti + 2H_2_O ⇌ [Ti(OH)_2_]^2+^ + 2H^+^ + 4e^−^ (2)

The oxygen reduction proceeds predominantly on the better-aerated surface outside the crevice:

O_2_ + 2H_2_O + 4e^−^ → 4OH^−^ (3)

The crevice interior restricts the transport of dissolved O_2_, thereby promoting spatial separation of the anodic and cathodic reactions. At the same time, Ti^4+^ species generated by anodic dissolution undergo hydrolysis within the confined crevice environment, producing H^+^ and thereby contributing to local acidification through reactions such as [56]:

Ti^4+^ + 2H_2_O ⇌ [TiOH_2_]^2+^ + 2H^+^ (4)

Ti^4+^ + 4H_2_O ⇌ Ti(OH_)4_ + 4H^+^ (5)

Concurrently, the protons facilitate the chemical dissolution of the oxide film [56]:

TiO_2_ + 2H^+^ ⇌ TiO^2+^ + H_2_O (6)

The reaction (6) is predominant at pH 1–12 above 1.8 V (Supplementary Figure S1). Thus, the restricted supply of O_2_ combined with the hydrolysis of dissolved Ti^4+^ species, promotes acidification within the crevice and inhibits local repassivation. Because the local pH within the crevice was not directly measured in the present study, the proposed acidification should be regarded as a possible mechanistic explanation based on the established crevice-corrosion mechanism rather than as direct experimental evidence from the present measurements.

The resulting accumulation of positively charged metal and protons drives chloride migration into the crevice to maintain electroneutrality. Consequently, the confined solution becomes progressively depleted in oxygen and enriched in H^+^ and Cl^−^, causing it to diverge from the neutral bulk pH and creating conditions sufficiently aggressive (pH <1.0 has been reported [57]) to destabilize the passive film and sustain localized corrosion [10,11,12]. As a result, titanium is in passive state (i.e., at a nobler potential) outside the crevice mouth and in active dissolution state (i.e., at a more negative potential) inside the crevice.

Typically, titanium undergoes spontaneous active dissolution in deoxygenated chloride media at pH < 2.3 [56]. Importantly, in the active state, Ti oxidizes to Ti^3+^ (Equation (7)), which is then hydrolyzed (Equation (8)):

Ti^0^ → Ti^3+^ + 3e^−^ (7)

Ti^3+^ + H_2_O ⇌ (TiOH)^2+^ + H^+^ (8)

Electrochemically active (TiOH)^2+^ species egress outside the crevice where they are subsequently oxidized to Ti(IV) at the passive surface:

H_2_O + (TiOH)^2+^ ⇌ [Ti(OH)_2_]^2+^ + H^+^ + e^−^ (9)

The [Ti(OH)_2_]^2+^ ions are transported back to the confinement of the crevice, where they are reduced to (TiOH)^2+^ at the active surface [56], which as a result will undergo the ennoblement of its potential and autopassivation. The necessary condition to autopassivation and spontaneous cessation of localized corrosion is the concentration of Ti(IV) reaching a critical value [56], which in turn is strongly dependent on geometric parameters of the crevice (length, width) and mass transport limitations.

In the light of the classical mechanism of autopassivation given above, it becomes evident that substrate condition can strongly influence this process. Compared with the conventionally processed alloy, the AMTi64 exhibited a greater tendency toward sustained localized attack once passive film breakdown initiated. Electrochemically, the AMTi64 alloy showed less evidence of effective repassivation, whereas the conventional alloy displayed a clear current decrease after the initial increase, suggesting recovery of passivity and metastable propagation of localized corrosion (Figure 5). Post-corrosion observations support the same interpretation: the AMTi64 substrate developed a large and rougher corroded area copiously covered by porous corrosion products, consistent with a more aggressive and sustained confined environment, while the conventional alloy showed a more superficial and laterally distributed attack without formation of a deep, well-defined cavity on the millimeter scale.

The overall greater susceptibility of the AMTi64 alloy to sustained localized attack under crevice conditions may be associated with its fine lamellar α + β microstructure, particularly the high density of α/β interfaces. These features can increase the kinetics of the reactions (2–8) leading to (i) an excessively aggressive crevice chemistry in terms of pH and Cl^−^ concentration and (ii) a massive clogging of the crevice with hydrolyzed species, which was observed by de Damborenea [50] and Mora-Sánchez [1,51] in AMTi64. As a result, both mass transport and repassivation are hindered: if Ti(IV) species cannot be delivered back to the innermost regions of the crevice in sufficient quantity to be reduced, autopassivation cannot occur. This explains why, while the bulk solution conditions were identical for both substrates, the AMTi64 microstructure made the localized breakdown process easier to initiate and more difficult to arrest [12,45,49].

Within this context, the role of hierarchical coating should be interpreted carefully. The multilayer PEO + PCL_SEAL_ + PLA_BF_ system was designed as a mitigation strategy to interfere with the processes required for establishing the critical crevice environment. However, a system with hierarchical porosity itself can and does contain nano-crevices. These are a result of the BF process method, where a polymer-volatile solvent solution (chloroform-based in the present case) is placed on the titanium alloy surface under 100% relative humidity conditions. As water vapor droplets condense on the solution surface, they act as a temporary template. After solvent evaporation, the polymer solidifies around these droplets and forms a honeycomb-like porous structure.

SEM observations from Figure 3 and Figure 4 reveal that the PEO layer and the polymer topcoat developed with essentially similar morphology on both alloys, suggesting that the substrate had no significant influence on the formation of the hierarchical coating architecture. Moreover, in some areas of Figure 4 where the PLA film is locally thinner and forms pool-like pores, the underlying porous PEO layer can still be distinguished under the PCL_SEAL_ layer. This provides evidence for the existence of potential nano-crevices in multilayer configuration of the hierarchical porous coating structure.

The fine breath-figure pores, with diameters ranging within 1–6 µm on ConvTi64 and within 1–5 µm on AMTi64, can provide pathways for electrolyte ingress into the outer porous polymer layer. However, these pores do not necessarily provide direct access to the metallic substrate, as electrolyte transport is additionally controlled by the underlying PCL sealing layer and the porous PEO coating. The comparable pore-size ranges observed on both substrates therefore suggest that the difference in corrosion behaviour (Figure 5) cannot be attributed to the size of the BF pores. Instead, electrolyte penetration and the possibility of reaching the metal/coating interface depend on the connectivity of the pores and the barrier function of the underlying layers. This interpretation is consistent with the electrochemical results, where the open porosity of the PLA breath-figure layer had little effect on the passive current when applied directly onto PEO, whereas PCL sealing produced a further approximately twofold reduction in passive current. Thus, although the BF pores facilitate electrolyte access into the hierarchical coating, the PCL and PEO layers limit further transport toward the substrate and thereby reduce the possibility of electrolyte reaching the metal/coating interface and initiating localized corrosion.

The PEO coating provides a firmly adherent ceramic barrier formed in situ (i.e., by electrochemical conversion) during anodizing of the substrate. However, because PEO-formed TiO_2_ is intrinsically porous, it cannot by itself fully eliminate mass transport pathways. It comprises an outer porous layer and an inner barrier layer which contains nano-voids, as seen in Figure 3d and elsewhere in the literature [1]. The subsequent PCL layer acts as a sealing phase, penetrating and partially blocking pores and microcracks in the oxide layer, thereby interrupting direct electrolyte access toward the metal/coating interface. Finally, the PLA breath-figure topcoat provides additional surface coverage and increases the tortuosity of mass transport at the coating/solution interface. Together, these layers reduce permeability, delay ingress of aggressive ions, and weaken the self-sustaining cycle of local acidification and chloride enrichment that drives crevice corrosion.

Gilbert et al. [58] demonstrated in modular hip prostheses retrieved for various reasons from 148 patients of different ages that crevice geometry, stress, micromotion and alloy coupling collectively determined the extent of in vivo corrosion. Rodrigues et al. [59] reported fretting-crevice corrosion mechanism in dental implants retrieved from patients suffering from peri-implantitis. Sutow et al. [60] observed no significant crevice attack on commercially pure Ti or Ti-6Al-4V ELI in deaerated Ringer’s solution at pH 7 and 37 °C when fretting was absent. Crevice dimensions are also critical: Zheng et al. [61] showed that reducing the gap size intensified localized corrosion of Ti-6Al-4V under simulated inflammatory conditions because stronger confinement restricted mass transport and promoted the development of aggressive crevice chemistry. Wu et al. observed the onset of crevice corrosion of ConvTi64 after 96 h of immersion in phosphate-buffered saline in the presence of 0.1 mm wide and 1.5 mm deep crevice [52].

Temperature and pH have generally been examined under substantially more severe conditions than those employed here. Kurtz et al. [62] reported progressively accelerated oxide degradation and phase-selective dissolution of Ti-6Al-4V with increasing temperature from 24 to 55 °C using an accelerated protocol involving cathodic activation and an H_2_O_2_-containing inflammatory solution, while Royhman et al. [63] found considerably greater corrosion charge and material loss at pH 3.0 than at pH 7.6 in a fretting-crevice model of a CoCrMo/Ti-6Al-4V hip taper. The limited studies addressing coated titanium under crevice-promoting conditions have further shown that coating interfaces may themselves become vulnerable sites: Reclaru et al. [64] detected crevice corrosion at the titanium–bone-cement interface and found vacuum-plasma-sprayed titanium surfaces less corrosion-resistant than anodized surfaces, whereas Otsuka et al. [65] attributed the reduced cyclic delamination life of plasma-sprayed hydroxyapatite coatings on Ti-6Al-4V in SBF partly to crevice corrosion and dissolution developing along the coating–substrate interface.

Against this background, the present study provides two distinct advances: first, it demonstrates that crevice corrosion of uncoated, heat-treated AMTi64 can be sustained at 37 °C in SBF with an initially near-physiological pH of 7.4, without fretting, added H_2_O_2_ or other oxidants, or prior acidification of the bulk electrolyte. At the same time, the present work also highlights why such coatings must be evaluated specifically under crevice-promoting conditions. Although hierarchical polymer/ceramic systems are often attractive because of their drug-delivery potential and biocompatibility, their multilayer nature introduces multiple interfaces and possible local heterogeneities. This means that a coating which performs well under freely exposed immersion does not necessarily guarantee equivalent behavior in a confined geometry. For this reason, the present study is significant not simply because it shows an improvement in corrosion resistance after coating but because it clarifies how a porous PEO-based hierarchical system behaves relative to a substrate that is already demonstrably prone to crevice corrosion under physiologically relevant conditions [19,32,39,65].

The post-coating polarization results (Figure 5 and Figure 8) clearly confirm that the hierarchical multilayer coating effectively protects the titanium alloy against corrosion under conditions that promoted localized attack in the uncoated substrate. This conclusion is further supported by the SEM observations of the AMTi64 alloy with the full coating system after corrosion testing in the crevice-free cell. Comparison of the coating morphology before testing (Figure 4) with that observed after potentiodynamic polarization (Figure 10) reveals localized alterations rather than complete preservation of the original structure. The accumulation of Na- and Cl-rich deposits within the larger pool-like pores indicates that these pores may locally retain the electrolyte and promote ion accumulation at coating interfaces. If such localized electrolyte retention persists during prolonged exposure, it could contribute to gradual degradation of the PCL sealing layer or interfacial regions and potentially increase the susceptibility of the coating to localized damage. Although the salt-rich deposits within the large pool-like pores and the round or elliptical PCL bubbles indicate local electrolyte access through defects in the PCL sealing layer and possible nano-crevices at the PCL_SEAL_ + PLA_BF_ interface, no crevice-type attack of the underlying Ti-6Al-4V substrate was detected. Thus, despite localized deformation and electrolyte penetration within the coating, the hierarchical system retained sufficient barrier functionality to prevent detectable propagation of crevice corrosion under the applied test conditions.

The EIS results further support the enhancement of the barrier properties provided by the hierarchical coating architecture. The lower resistance of the PEO layer is consistent with its intrinsically porous nature and the presence of electrolyte-accessible pathways through its outer porous structure. Importantly, the incorporation of the PCL layer and the subsequent addition of the PLA breath-figure layer sealed the pores in the ceramic layer and significantly increased their resistance, indicating that the sealing phase effectively restricted the electrolyte transport through the pores and microcracks of the underlying PEO layer. The shift in the barrier layer response of the PEO to much lower frequencies may be attributed to the much increased tortuosity of electrolyte transport pathways. These EIS results are consistent with the PDP response, where the PEO layer substantially reduced the passive current, while the addition of the sealing layer produced a further reduction in passive current for the complete hierarchical system. Overall, the EIS results indicate that the enhanced corrosion protection is primarily associated with the sealing effect of the PEO pores with polymer layer, which increases transport resistance and limits electrolyte penetration toward the AMTi64 substrate.

Considering the approximately 30 μm total thickness of the PEO + PCL_SEAL_ + PLA_BF_ system (Figure 4), a nominal crevice gap of approximately 10 μm at the coating surface corresponds to a total distance of approximately 40 μm between the opposing surface and the Ti-6Al-4V substrate. Thus, unlike the uncoated alloy, the metallic substrate was not directly exposed to the narrow gap associated with sustained crevice attack, while the multilayer coating further increased the transport distance and tortuosity of the electrolyte pathway. The Na- and Cl-rich deposits detected beneath the PCL layer (Figure 10 and Table 8) provide indirect evidence of electrolyte penetration and local ion accumulation within the confined interfacial regions. Although the local pH could not be measured, these observations are consistent with the development of localized crevice chemistry and chloride enrichment, but do not directly demonstrate acidification.

The present findings demonstrate that, for uncoated Ti-6Al-4V, and especially for the AMTi64 alloy, this local collapse can occur in SBF at 37 °C when the crevice size falls to a critical threshold of approximately ≤11 µm. The hierarchical coating improves the corrosion resistance by delaying or weakening the development of this critical confined chemistry. Accordingly, the concept of a critical crevice condition is central to understanding and designing fail-proof long-term corrosion protection strategies for titanium-based biomedical systems.

## 5. Conclusions

- Crevice corrosion of the uncoated wrought and DMLS Ti-6Al-4V substrates in SBF occurs even at 37 °C, showing that highly aggressive bulk conditions such as elevated temperature or initially acidic pH are not essential prerequisites for attack.
- Once the crevice gap reaches a critical sub-11 µm range, the local chemistry inside the crevice becomes sufficiently severe to destabilize the passive film. Sustained localized corrosion up to 79 μm in depth occurred in DMLS Ti-6Al-4V. In case of wrought Ti-6Al-4V alloy a recovery of passivity and metastable propagation of localized corrosion was observed.
- Despite the intrinsically multilayered nature of the hierarchical PEO + PCL_SEAL_ + PLA_BF_ coating system and the possible existence of natural interlayer gaps, the hybrid architecture was able to protect the substrate even under crevice-corrosion-prone conditions. The coated system showed improved corrosion resistance compared with the uncoated substrate due to the increase in the resistance of polymer-filled PEO pores and the tortuosity of the electrolyte pathways that delayed the response of the barrier layer.
- The onset of crevice corrosion in Ti-6Al-4V is governed by the combined effects of crevice geometry, local chemistry, substrate processing condition, and coating architecture.
- These findings are particularly important for biomedical titanium systems, where near-physiological environments cannot automatically be assumed to be safe from localized corrosion. Designing effective protection strategies therefore requires not only improving general barrier performance but also controlling the conditions that lead to the establishment of a critical crevice environment.

## Figures and Tables

**Table 1:** Local EDS analysis (at.%) of conventional (ConvTi64) and DMLS (AMTi64) substrates as per locations in Figure 2.

Specimen/Location		Mg	Al	Si	Ti	V	Fe
ConvTi64	Area	-	9.6	-	87.3	3.1	-
	1	0.4	7.8	0.5	88.0	3.0	0.1
	2	0.2	7.3	0.8	88.0	3.6	0.2
AMTi64	3	0.15	6.2	0.1	90.0	3.0	0.2
	4	0.15	6.4	0.1	90.0	3.0	0.1
	5	0.16	7.0	0.4	89.0	3.0	0.2

**Table 2:** PEO coating porosity estimated from image analysis of SEM micrographs of Figure 3.

Specimen	Pore Population Density, mm^−2^	Average Pore Size, µm	Porosity, %	Coating Thickness, µm
AMTi64-60s	1.32 × 10^6^	0.8	9.7	4.8
ConvTi64-60s	0.90 × 10^6^	1.1	13.1	5.2

**Table 3:** Local EDS analysis (at.%) of PEO coatings on ConvTi64 and AMTi64 alloys as per locations in Figure 3.

Specimen/Location			Na	Mg	Al	Si	P	S	Ti	Ca	Zn	O	V
AMTi64-60s	Plan	1	-	1.3	1.7	3.5	7.5	0.3	26.7	9.4	0.9	47.5	1.3
ConvTi64-60s		1	0.9	1.3	1.4	3.7	8.0	0.2	25.4	10	1	46.9	1.2
AMTi64-60s	Cross section	1	0.2	1.2	1.8	1.8	5.2	0.2	11.2	4.4	3.3	72.8	0.4
		2	0.3	0.4	2.6	1.3	2.9	0.7	19.9	1.3	0.7	69.8	0.7
ConvTi64-60s		1	0.6	1.5	1.4	3.5	5.8	-	11.0	7.3	0.2	68.0	0.7
		2	0.4	-	2.8	1.0	3.4	1.5	22.5	0.4	0.1	67.3	0.6

**Table 4:** Local EDS analysis (at.%) of ConvTi64 with PEO + PCL_SEAL_ + PLA_BF_ coating as per Figure 4c.

Analysis Location	C	Al	Mg	Ca	P	Si	Ti	V	Zn	O	S
Figure 4c	38.0	1.6	0.6	2.0	3.6	0.3	18.9	0.7	0.9	32.9	0.4

**Table 5:** Passive current densities, corrosion and crevice potentials for AMTi64 and ConvTi64with and without PEO + PCL_SEAL_ + PLA_BF_ coating in a crevice cell.

Sample	i_pass_ (µA cm^−2^)	E_corr_ (V_Ag/AgCl_)	E_crev_ (V_Ag/AgCl_)
ConvTi64 with coating	0.09	0.379	-
AMTi64 with coating	0.15	0.366	-
ConvTi64	0.76	−0.363	1.70
AMTi64	0.96	−0.246	1.70

**Table 6:** Local EDS analysis (at.%) of AMTi64 and ConvTi64 as per locations in Figure 6.

	Location	O	Na	Al	Mg	P	Si	Cl	Ca	Ti	V
ConvTi64	1	40.6	0.8	5.8	-	-	0.6	0.36	0.22	51.1	2.55
	2	-	-	9.3	-	-	-	-	-	85.4	5.3
	3	59.2	1.6	2.3	-	-	0.2	0.3	0.6	33.6	1.9
AMTi64	1	33.1	0.5	5.8	-	0.4	-	-	-	58.4	1.9
	2	65.6	1.2	1.2	0.3	3.3	-	0.2	1.2	25.6	1.1
	3	61.7	1.1	2.5	-	1.8	-	-	0.5	31.0	1.2

**Table 7:** Fitted EEC parameters for EIS spectra of PEO coaiting on AMTi64 with succesive polymeric layers.

Coating	CPE_bf_, μS s^n^ cm^−2^	n_bf_	R_bf_, kΩ cm^2^	CPE_por_, μS s^n^ cm^−2^	n_por_	R_por_, kΩ cm^2^	CPE_b_, μS s^n^ cm^−2^	n_b_	R_b_, kΩ cm^2^
PEO + PLA_BF_	2.3	0.73	1.12	1.98	0.80	5.01	21.52	0.57	10,000
PEO + PCL_SEAL_ + PLA_BF_	4.2	0.65	0.75	1.29	0.87	3.45	50.93	0.52	10,000

**Table 8:** Local EDS analysis (at.%) of AMTi64 with PEO + PCL_SEAL_ + PLA_BF_ coating as per locations in Figure 10.

Location	O	Na	Mg	Al	Si	P	S	Cl	K	Ca	Ti	V	Zn
1	88.7	1.6	0.5	0.8	0.3	2.3		0.5		1.3	4.2		
2	10.2	41.8						47.6	0.1		0.4		
3	62.0		1.0	2.5	0.4	5.8	0.3	0.2		3.3	22.5	1.0	1.1
4	81.9	2.0	0.6	1.5	0.2	3.3		0.4		1.5	8.2	0.4	
5	10.6	46.5				0.2		41.8		0.2	0.7		
6	19.2	38.3	0.5	1.0	0.2	2.9		28.7		2.0	6.8	0.3	0.2

## Data Availability

All data presented in this work will be made available through Docta Complutense repository https://docta-ucm-es.bucm.idm.oclc.org/handle/20.500.14352/16 (accessed on 1 October 2026).

## Supplementary Materials

The following supporting information can be downloaded at https://www.mdpi.com/article/10.3390/jfb17100508/s1, Figure S1: Predominance potential-pH diagram of Ti species. Table S1: Fitted EEC parameters for EIS spectra of AM Ti-6Al-4V substrate after 1 h of immersion in SBF at 37 °C. Table S2: Fitted EEC parameters for EIS spectra of flash-PEO coated AM Ti-6Al-4V substrate after 1 h of immersion in SBF at 37 °C.

## Abbreviations

The following abbreviations are used in this manuscript:

AM	Additive Manufacturing
BF	Breath figure
DMLS	Direct Metal Laser Sintering
EBM	Electron Beam Melting
EDS	Energy Dispersive Spectroscopy
EEC	Equivalent electrical circuit
HA	Hydroxyapatite
I_RMS_	Root Mean Square Current
L-PBF	Laser Powder Bed Fusion
OCP	Open Circuit Potential
PCL	Polycaprolactone
PDP	Potentiodynamic Polarization
PEO	Plasma Electrolytic Oxidation
PLA	Polylactic Acid
SBF	Simulated Body Fluid
SEM	Scanning Electron Microscopy
SLM	Selective Laser Melting
TRIS	Tris(hydroxymethyl)aminomethane
WCA	Water Contact Angle
XRD	X-Ray Diffraction
