# Supplementary material for: Crevice Corrosion Mitigation in Ti-6Al-4V DMLS Alloy via Hybrid PEO/PCL/PLA Hierarchical Coatings for Biomedical Implants
Source: J Funct Biomater. 2026 Oct 8;17(10):508. doi: 10.3390/jfb17100508 (PMC13706034; doi:10.3390/jfb17100508)

Crevice Corrosion Mitigation in Ti–6Al–4V DMLS Alloy
via Hybrid PEO/PCL/PLA Hierarchical Coatings for Biomedical Implants

Shaghayegh Javadi [1,*], Enrique Martinez-Campos [2,3], Raúl Arrabal [1] and Endzhe Matykina [1,3,*]

[1] Departamento de Ingeniería Química y de Materiales, Facultad de Ciencias Químicas, Universidad
Complutense de Madrid, 28040 Madrid, Spain; rarrabal@ucm.es
[2] Instituto de Ciencia y Tecnología de Polímeros (ICTP), Consejo Superior de Investigaciones Científicas (CSIC), C/Juan de la Cierva 3, 28006 Madrid, Spain; e.martinez.campos@csic.es
[3] Unidad Asociada al ICTP, IQM (CSIC), Grupo de Síntesis Orgánica y Bioevaluación, Instituto
Pluridisciplinar (UCM), Paseo de Juan XXIII, 1, 28040 Madrid, Spain
* Correspondence: shjavadi@ucm.es (S.J.); e.matykina@quim.ucm.es (E.M.)

SUPPLEMENTARY MATERIAL

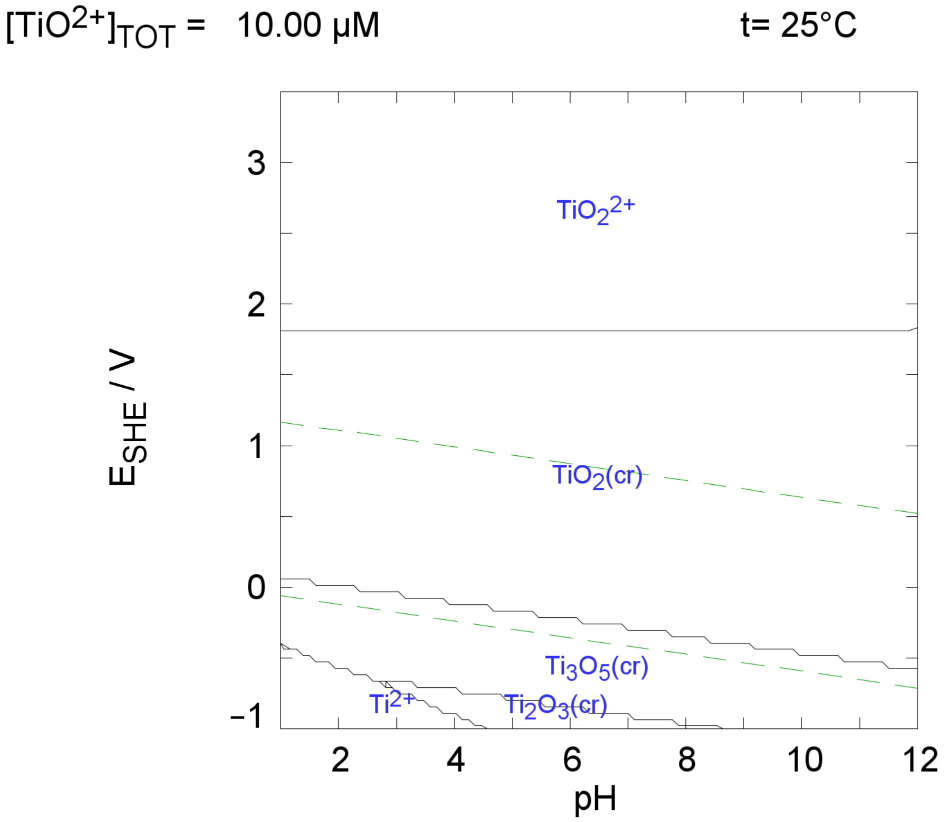

Supplementary Figure S1. Predominance potential-pH diagram of Ti species.

Supplementary Table S1. Fitted EEC parameters for EIS spectra of AM Ti-6Al-4V substrate after 1 h of immersion in SBF at 37°C.

CPE_{por}, μSs^ncm^{-2}	n_{por}	R_{por}, $k\Omega cm^2$	CPE_b, μSs^ncm^{-2}	n_b	R_b, $k\Omega cm^2$
17.23	0.87	0.038	13.37	0.85	4135

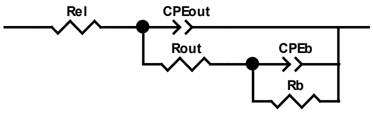

Supplementary Table S2. Fitted EEC parameters for EIS spectra of flash-PEO coated AM Ti-6Al-4V substrate after 1 h of immersion in SBF at 37°C.

CPE_{port}, μSs^ncm^{-2}	n_{por}	R_{por}, $k\Omega cm^2$	CPE_{sc}, μSs^ncm^{-2}	n_{sc}	R_{sc}, $k\Omega cm^2$	CPE_b, μSs^ncm^{-2}	n_b	R_b, $k\Omega cm^2$
9.84	0.62	0.025	18.65	0.76	1.51	18.77	0.83	403

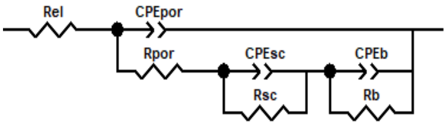

Supplement: Supplementary file 1 [file jfb-17-00508-s001.zip › jfb-4598075-supplementary.pdf]
